# Potential Clinical Roles for Metabolic Imaging with Hyperpolarized [1-^13^C]Pyruvate

**DOI:** 10.3389/fonc.2016.00059

**Published:** 2016-03-11

**Authors:** Eva M. Serrao, Kevin M. Brindle

**Affiliations:** ^1^Li Ka Shing Centre, Cancer Research UK Cambridge Institute, University of Cambridge, Cambridge, UK; ^2^Department of Biochemistry, University of Cambridge, Cambridge, UK

**Keywords:** cancer, metabolism, imaging, hyperpolarized, pyruvate, therapy monitoring

Clinical oncology relies increasingly on biomedical imaging, with anatomical imaging, especially using CT and ^1^H-MRI, forming the mainstay of patient assessment, from diagnosis to treatment monitoring. However, the need for further improvements in specificity and sensitivity, coupled with imaging techniques that are reaching their limit of clinically attainable spatial resolution, has resulted in the emergence and growing use of imaging techniques with additional functional readouts, such as ^18^FDG-PET and multiparametric MRI. These techniques add a new dimension to our understanding of the biological behavior of tumors, allowing a more personalized approach to patient management.

An important functional imaging target in cancer is metabolism. PET measurements of ^18^Fluorodeoxyglucose uptake (^18^FDG-PET), a ^18^F-labeled glucose analog, and ^1^H-MRS measurements, have both been used to investigate tumor metabolism for diagnostic purposes. However, clinical applications of MRS have been hampered by low sensitivity and consequently low spatial and temporal resolution (1). Nuclear spin hyperpolarization of ^13^C-labeled substrates, using dynamic nuclear polarization (DNP), which radically increases the sensitivity of these substrates to detection by ^13^C MRS (2), has created a renewed interest in MRS measurements of tissue metabolism. Successful translation of this technique to the clinic was achieved recently with measurements of [1-^13^C]pyruvate metabolism in prostate cancer (3) (see Figure 1). We explore here the potential clinical roles for metabolic imaging with hyperpolarized [1-^13^C]pyruvate.

**Figure 1:**
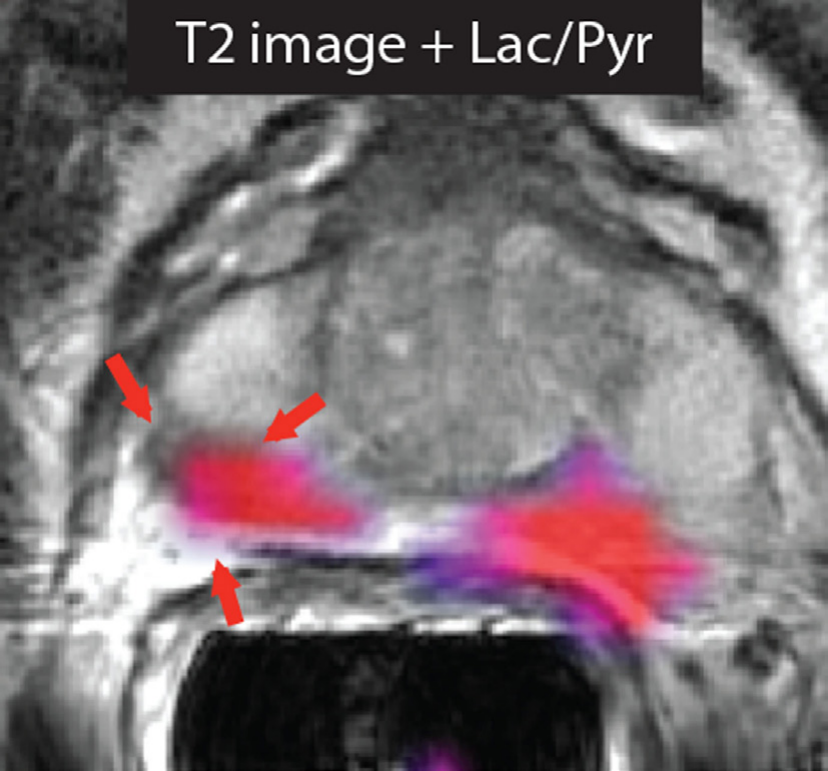
**Imaging prostate cancer with hyperpolarized [1-^13^C]pyruvate**. The T_2_-weighted image of a patient with biopsy-proven bilateral prostate cancer showed only a unilateral decrease in signal intensity. However, the metabolic image ([1-^13^C]lactate/[1-^13^C]pyruvate ratio) detected disease on both the right and left sides of the prostate. Reproduced from Nelson et al. (3) with permission.

## Dynamic Nuclear Polarization

Dynamic nuclear polarization, which can increase the signal-to-noise ratio in the solution-state ^13^C MR experiment by 10^4^- to 10^5^-fold (4), has enabled *in vivo* imaging of various metabolites and their enzymatic conversion into other species, as well as metabolic fluxes in central metabolic pathways, such as glycolysis (5–7) and the tricarboxylic acid cycle (8–10). The principle limitation of the technique is the short half-life of the polarization; for [1-^13^C]pyruvate *in vivo*, this is typically between 30 and 40 s, which means that the hyperpolarized signal will last for 2–3 min. Therefore, the substrate, whose metabolism is to be imaged, must be transferred promptly from the polarizer, injected intravenously, and then transit quickly *via* the circulation to the tissue of interest, where it should be taken up and metabolized rapidly (11, 12). To date, numerous molecules, in addition to ^13^C-labeled pyruvate, have been successfully hyperpolarized and their metabolism imaged, including [1,4-^13^C_2_]fumarate, as a marker of cell necrosis (13, 14); [U-^2^H, U-^13^C]glucose for assessment of glycolytic and pentose phosphate pathway activities and for detecting early treatment response (7); ^13^C-labeled bicarbonate for *in vivo* mapping of pH (15); and ^13^C-labeled urea as a marker of perfusion (16), among others (13,  16–18). Despite initial interest in vascular imaging (19–21), the main focus has been on imaging metabolism in tumors (13, 17, 22) and cardiac tissue (23–27).

## Pyruvate

Pyruvate is an important intermediate in many biochemical pathways (28). As an end product of glycolysis, pyruvate can be reduced by NADH to generate lactate, in the readily reversible reaction catalyzed by lactate dehydrogenase, or transaminated by glutamate, in the reversible reaction catalyzed by alanine aminotransferase (ALT), to form alanine. In tissues with high levels of mitochondrial activity, such as heart muscle, pyruvate may be irreversibly decarboxylated to form carbon dioxide in the reaction catalyzed by the mitochondrial pyruvate dehydrogenase (PDH) complex (26). Since increased aerobic glycolysis is a well-recognized hallmark of cancer (29, 30), this has made it an attractive pathway to probe for diagnostic and treatment monitoring purposes (3, 17, 31).

### Potential Clinical Roles

Preclinical studies have demonstrated that hyperpolarized [1-^13^C]pyruvate is a promising probe for oncological imaging, with increased lactate labeling observed in tumors as compared to normal tissues (31, 32). The substrate has the potential to be used in many steps of patient management. A recent study demonstrated the potential of hyperpolarized [1-^13^C]pyruvate as an imaging biomarker for early detection and secondary screening of pancreatic cancer, where a decrease in the hyperpolarized [1-^13^C]alanine/[1-^13^C]lactate ratio was observed in the progression from precursor lesions to adenocarcinoma (33). In another study, [1-^13^C]pyruvate detected metabolic changes prior to tumor formation (34). Additionally, in the first reported clinical trial in prostate cancer, increased lactate labeling was observed in histologically confirmed areas of disease that were not identifiable by conventional ^1^H-MRI measurements (3) (Figure 1). The few studies that have explored the role of [1-^13^C]pyruvate in grading and prognosis, which were in the transgenic mouse model of prostate adenocarcinoma (TRAMP), have also produced promising results (35, 36). Tumor grading by biopsy can sometimes be difficult depending on the accessibility of the organ of interest. Translation of the DNP technique to the clinic may allow more accurate targeting of biopsy procedures. Since lactate labeling is increased in regions of hypoxia, the technique also has the potential to be used for treatment planning in radiotherapy (37, 38). Clinical assessments of tumor responses to treatment are still based largely on observed changes in tumor size (39). However, this might not always be appropriate, particularly for detection of early responses or if the drug does not result in tumor shrinkage, for example, in the case of antiangiogenic drugs (40, 41). Additionally, treatment assessment using ^18^FDG-PET is difficult in some organs, e.g., prostate and brain, due to both low tumor uptake and increased background uptake, respectively (40). Evaluation of treatment response is likely to be the clinical scenario where hyperpolarized [1-^13^C]pyruvate will have the most impact, as it could lead to immediate changes in clinical management, allowing the clinician to change a non-responding patient to a more effective drug at an early stage (40). Early assessment of treatment response could also be used to accelerate the introduction of new drugs into the clinic by providing an indication of drug efficacy in early stage clinical trials. In support of this are numerous studies showing early decreases in hyperpolarized ^13^C-labeled exchange between injected [1-^13^C]pyruvate and the endogenous lactate pool in a range of cancer models following treatment with cytotoxic chemotherapy (17, 42), targeted drugs (43–45), and radiotherapy (41, 46, 47).

There is as yet no direct evidence to support the suggestion that residual disease/recurrence can be identified by increased lactate labeling. However, observations of increased lactate labeling in areas of disease and following disease progression (3, 33) make this likely. There is, however, evidence that hyperpolarized [1-^13^C]pyruvate can be used to assess normal tissue toxicity, with an increase in the [1-^13^C]lactate/[1-^13^C]pyruvate ratio occurring in radiation-induced lung injury (48, 49).

## Advantages of Metabolic Imaging with [1-^13^C]Pyruvate

### Advantages Compared to ^18^FDG-PET

Metabolic imaging of cancer in the clinic has principally been with ^18^FDG-PET, which has been used to stage tumors and to assess treatment response. Despite its high ­sensitivity and capability to provide whole-body images, the use of ionizing radiation is a drawback, limiting its application in children and women of reproductive age, and when multiple investigations are needed, for example, as might be required for guiding treatment in an individual patient. A similar clinical role can be envisaged for [1-^13^C]pyruvate as has been established for ^18^FDG-PET. Both techniques can be used to detect increased glycolytic flux (50) and have been shown to be comparably sensitive in detecting tumor response to treatment (51). However, since hyperpolarized [1-^13^C]pyruvate effectively detects lactate accumulation (11), a defining feature of cancer metabolism, i.e., the failure to oxidize pyruvate in the presence of oxygen and reduce it instead to lactate (the “Warburg Effect”), this means that hyperpolarized [1-^13^C]pyruvate may be more specific for detecting cancer than ^18^FDG-PET. The latter detects only elevated levels of glucose uptake, which is a feature of many normal tissues and cancer, for example, the brain. The specificity of cancer detection by hyperpolarized [1-^13^C]pyruvate may be confounded, however, by the presence of hypoxia, which will also lead to lactate accumulation and increased lactate labeling (38). Another drawback of imaging with hyperpolarized [1-^13^C]pyruvate is that the short half-life of the polarization precludes whole-body imaging.

### Advantages Compared to ^1^H MRS

^1^H-MR spectroscopy and spectroscopic imaging measurements of tissue metabolite profiles are label-free and have found some applications, for example, in identifying different types of brain tumor (52). A notable example is the detection of 2-hydroxyglutarate, which can be used to identify glioblastomas with isocitrate dehydrogenase mutations (IDH) (53). ^1^H MRSI has also proved to be important in the prostate, where it can improve the specificity of detection and determination of tumor extent when combined with other MR imaging sequences (54). However, detectable metabolites are present in only millimolar concentrations, as compared to tissue water protons, which are present at ~80M, which results in long data acquisition times and limited spatial resolution. In addition, data processing can be more complex and the biochemical information provided may be unfamiliar to many clinicians, which has limited routine clinical application. Moreover, ^1^H MR spectroscopy and spectroscopic images of metabolite profiles provide a static picture of tumor metabolism. On the other hand, imaging with hyperpolarized ^13^C-labeled substrates provides dynamic metabolic flux information in the form of images that can be acquired at relatively high spatial and temporal resolutions and therefore should provide an improved assessment of tumor behavior. Additionally, coinjection of different hyperpolarized substrates could also provide additional functional information in the same acquisition, e.g., pyruvate for assessing glycolytic activity and urea for assessing tumor perfusion (55).

## Combining Metabolic Imaging with Hyperpolarized [1-^13^C]Pyruvate with New Technologies

### PET-MRI

This is an emerging combined imaging modality with significant potential for clinical assessment of cancer patients (56). Simultaneous PET-MR measurements with hyperpolarized ^13^C- and ^18^F-labeled substrates would allow a multiparametric assessment of the primary lesion and its metastases in a single imaging session, which potentially could be used to identify imaging-based phenotypes that have prognostic value and which may give a more specific readout of treatment response. For example, PET measurements of ^18^FDG uptake assess just the first three steps in tumor glucose metabolism, i.e., delivery *via* the bloodstream, uptake on the glucose transporters, and phosphorylation and trapping in the reaction catalyzed by hexokinase. ^13^C MRSI measurements of the exchange of hyperpolarized ^13^C-labeled exchange between injected [1-^13^C]pyruvate and the endogenous lactate pool again assess delivery *via* the bloodstream and effectively the last two steps in the glycolytic pathway, i.e., the steps catalyzed by lactate dehydrogenase and the plasma membrane monocarboxylate transporters. Therefore, by combining ^18^FDG-PET and hyperpolarized [1-^13^C]pyruvate measurements, we may be able to assess flux in the entire glycolytic pathway, for example, increased mitochondrial oxidation of pyruvate may have no effect on ^18^FDG uptake but could decrease ^13^C labeling of lactate. There are other PET probes of tumor metabolism that could also be used alongside hyperpolarized [1-^13^C]pyruvate, and which could provide complementary information. These include ^11^C-acetate, as a marker of fatty acid synthesis, and labeled glutamine, which can be used to assess glutaminolysis; both of which are upregulated in tumor cells (57, 58). These PET probes may be especially useful in tumors where ^18^FDG is ineffective, e.g., in prostate tumors (59) and in gliomas (60), and where the corresponding hyperpolarized ^13^C-labeled probes are limited. For example, the metabolism of hyperpolarized [1-^13^C]acetate has been detected *in vivo* (61); however, the short lifetime of the hyperpolarization means that it could not be used to monitor fatty acid synthesis, where PET studies with [1-^11^C]acetate in animal tumor models have shown that it can take over 60 min before there is substantial incorporation into the fatty acid pool (62). In the case of [5-^13^C]glutamine, a relatively short hyperpolarization lifetime and slow metabolism (63) has precluded imaging *in vivo* (64).

### Liquid Biopsies

Blood and urine biomarkers, obtained from “liquid biopsies,” are also evolving, providing information in a non-invasive way allied to the advantages of collection simplicity and relatively low cost. Many body fluid biomarkers have been reported for different types of cancer; however, few have become established in the clinic, usually because they lack specificity. A recent promising example is a panel of three urine biomarkers for early detection of pancreatic cancer (65). Rapid advances in DNA sequencing technology have allowed somatic mutations present in tumor cells to be detected and tracked in blood-borne circulating tumor DNA (ctDNA). These fragments of DNA, which have been detected with most types of cancer, have been demonstrated to have potential roles in early detection, staging, and in detecting response to therapy and acquired resistance to treatment (66, 67). Although measurements with hyperpolarized ^13^C-labeled cell substrates and these new circulating biomarkers are still their infancy it seems likely that they will provide complementary information, for example, in the assessment of tumor heterogeneity.

## Conclusion and Future Directions

Imaging with hyperpolarized ^13^C-labeled cell substrates has the potential to become a powerful tool in many steps of clinical evaluation, allowing a more personalized approach to treatment. The first clinical trial established the feasibility of imaging human tumors with hyperpolarized [1-^13^C]pyruvate. Since this substrate can be used to assess glycolysis, which is upregulated in many tumors, then this should make it a very general tool for oncological imaging in the clinic. Despite the biological insights that imaging with hyperpolarized ^13^C-labeled substrates promises to deliver in the clinic, it will nevertheless have to prove itself against established and emerging clinical techniques, demonstrating that it can provide unique information that changes clinical practice.

## Author Contributions

Both authors listed, have made substantial, direct, and intellectual contribution to the work, and approved it for publication.

## Conflict of Interest Statement

KB’s lab has a research agreement with GE Healthcare (GEH) and holds patents on DNP technology with GEH.

## Abbreviations

^18^FDG, ^18^Fluorodeoxyglucose; MRS, magnetic resonance spectroscopy; MRSI, magnetic resonance spectroscopic imaging; PET, positron emission tomography.
